# Effectiveness of high-frequency oscillatory ventilation for the treatment of neonatal meconium aspiration syndrome

**DOI:** 10.1097/MD.0000000000017622

**Published:** 2019-10-25

**Authors:** Li-xia Hao, Fei Wang

**Affiliations:** a Department of Neonatology; b Department of Ophthalmology, Yan’an University Affiliated Hospital, Yan’an, China.

**Keywords:** effectiveness, high-frequency oscillatory ventilation, neonatal meconium aspiration syndrome, randomized controlled trial, safety

## Abstract

**Background:**

The purpose of this study is to raise nonjudgmental awareness and attention to current experience of high-frequency oscillatory ventilation (HFOV) for the treatment of neonatal meconium aspiration syndrome (NMAS).

**Methods:**

We will comprehensively search literature from the databases of Cochrane Library, PubMed, Embase, Web of Science, WorldSciNet, PsycINFO, Allied and Complementary Medicine Database, Chinese Biomedical Literature Database, and China National Knowledge Infrastructure from inception until July 1, 2019 without language limitation. We will also handle searching the bibliographies of all relevant studies found for unpublished literatures. Statistical analysis will be conducted using RevMan 5.3 software.

**Results:**

The outcomes include function inhaled oxygen concentration, oxygenation index, arterial oxygen tension/alveolar arterial oxygen tension, partial pressure of oxygen, partial pressure of carbon dioxide, transcutaneous arterial oxygen saturation, duration of hospitalization, and adverse events.

**Conclusion:**

This study will provide an exhaustive view of HFOV for treating infants with NMAS.

**PROSPERO registration number:**

PROSPERO CRD42019140520.

## 1 Introduction

Neonatal meconium aspiration syndrome (NMAS) is a very serious disease.^[1–3]^ It often occurs prior, during or right after the delivery.^[4–7]^ Although it is often not life-threatening, it can lead to some several health complications for the infants.^[8–10]^ If such condition is severe or untreatable, it is very fatal for the infants.^[8–12]^ It often happens when the infants experience stress from pregnancy mothers that goes past the due date for more than 4 weeks, difficulty delivery or long labor, infection, and certain health problems experienced by the pregnant mother.^[13–18]^ If it occurs, infants often manifest as rapidly breath or grunt during breathing, or even stop breathing, and may also follows the symptoms of cyanosis, limpness, and low blood pressure.^[4–6,15–17]^ High-frequency oscillatory ventilation (HFOV) has been reported to treat such condition very effectively.^[1,9,18–21]^ However, there is still insufficient evidence to support it on the evidence-based medicine level. Therefore, this study will investigate the effectiveness and safety of HFOV for the treatment of infants with NMAS.

## 2 Methods

### 2.1 Eligibility criteria for study selection

#### 2.1.1 Types of studies

We intend to include randomized controlled trials (RCTs) of HFOV for the treatment of NMAS. We will include Non-RCTs and quasi-RCTs, and case studies.

#### 2.1.2 Types of interventions

The intervention measures taken by the experimental group must be HFOV.

The control group receives any different treatments from the experimental group.

#### 2.1.3 Types of participants

Participants with a clinical diagnosis of NMAS will be included with no restrictions of race, gender, country, and sources.

#### 2.1.4 Types of outcome measurements

The outcomes consist of function inhaled oxygen concentration, oxygenation index, arterial oxygen tension/alveolar arterial oxygen tension, partial pressure of oxygen, partial pressure of carbon dioxide, transcutaneous arterial oxygen saturation, duration of hospitalization, and adverse events.

### 2.2 Search strategy

#### 2.2.1 Electronic databases sources

We intend to carry out a literature search from Cochrane Library, PubMed, Embase, Web of Science, WorldSciNet, PsycINFO, Allied and Complementary Medicine Database, Chinese Biomedical Literature Database, and China National Knowledge Infrastructure from inception until July 1, 2019 without language limitation. During literature retrieval, information experts have provided help and guidance. To fully retrieve qualified studies, a comprehensive search strategy for Cochrane Library is shown in Table 1. We will also adapt identical search strategies to other electronic databases.

**Table 1:**
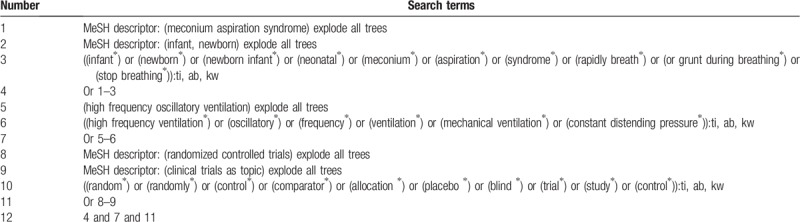
Search strategy used in Cochrane Library database.

#### 2.2.2 Other literature sources

We will also retrieve unpublished literatures, including dissertations, ongoing trials, and conference abstracts.

### 2.3 Study selection

The literature selection will be independently performed by 2 researchers. This study consists of 2 stages. First, we will make a preliminary selection by scanning the titles and abstracts. Second, we will read all remaining relevant studies for further selection based on the eligibility criteria. If there are difference opinions between 2 researchers, a 3rd researcher will help to reach an agreement by discussion. The study selection process is presented in the flowchart.

### 2.4 Data extraction

We will investigate the characteristics of different clinical trials qualitatively. All data extraction will be conducted by 2 researchers independently. Any different opinions between 2 researchers, a 3rd researcher will help to reach a consensus through discussion. The main extraction information consists of title, primary author, publication time, country, patients, study design, study methods, treatment details, outcomes, safety, and follow-up details.

### 2.5 Risk of bias assessment

Two researchers will assess risk of bias independently using Cochrane risk of bias tool based on the Cochrane Handbook for Systematic Review of Interventions. Assessment items include 7 aspects. If there are different opinions, a 3rd researcher will help to make the final decision by discussion.

### 2.6 Measurements of treatment effect

Continuous variables will be expressed as mean difference or standardized mean difference and 95% confidence intervals. Categorical variables will be calculated as risk ratio and 95% confidence intervals.

### 2.7 Heterogeneity assessment

Heterogeneity will be checked based on the *I*^2^ test. *I*^2^ ≤ 50% indicates low heterogeneity, and a fixed-effects model is used. On the contrary, *I*^2^ > 50% indicates significant heterogeneity, and a random-effects model is utilized.

### 2.8 Data synthesis

Statistical analysis will be performed using RevMan 5.3 software. If there is low heterogeneity (*I*^2^ ≤ 50%), meta-analysis will be conducted if more than 2 studies reporting an outcome have similar study design, patients, methods, and outcomes. If there is high heterogeneity (*I*^2^ > 50%), we will carry out subgroup analysis. If a meta-analysis still cannot be performed after subgroup analysis, a comprehensive narrative summary will be reported to describe the studies.

### 2.9 Subgroup analysis

We will carry out subgroup analysis to explore the source of heterogeneity according to the different treatments, comparators, and outcomes.

### 2.10 Sensitivity analysis

We will conduct sensitivity analysis to check stability of outcome results by removing low quality RCTs.

### 2.11 Publication bias

The potential publication bias of all studies will be evaluated by funnel plot and Egger regression test.^[22,23]^

### 2.12 Ethics and dissemination

The findings of this study based on the published evidence; thus, ethical approval is not inquired. We intend to publish this study at peer-reviewed journals.

## 3 Discussion

This study aims to systematically assess the effectiveness and safety of HFOV for the treatment of NMAS. It will provide a detailed and evidence-based overview of the effectiveness of function inhaled oxygen concentration, oxygenation index, and arterial oxygen tension/alveolar arterial oxygen tension, partial pressure of oxygen, partial pressure of carbon dioxide, transcutaneous arterial oxygen saturation, and duration of hospitalization. Results of this study will provide evidence base to the clinical practice for selecting HFOV for infants with NMAS.

## Author contributions

**Conceptualization:** Li-xia Hao, Fei Wang.

**Data curation:** Li-xia Hao, Fei Wang.

**Formal analysis:** Li-xia Hao, Fei Wang.

**Investigation:** Fei Wang.

**Methodology:** Li-xia Hao.

**Project administration:** Fei Wang.

**Resources:** Li-xia Hao.

**Software:** Li-xia Hao.

**Supervision:** Fei Wang.

**Validation:** Li-xia Hao, Fei Wang.

**Visualization:** Li-xia Hao, Fei Wang.

**Writing – original draft:** Li-xia Hao, Fei Wang.

**Writing – review & editing:** Li-xia Hao, Fei Wang.
